# Data on RNA-seq analysis of the cocoa pod borer pest *Conopomorpha cramerella* (Snellen) (Lepidoptera: Gracillariidae)

**DOI:** 10.1016/j.dib.2020.106638

**Published:** 2020-12-09

**Authors:** Nor Azlan Nor Muhammad, Intan Azlinda Ramlee, Diana Mohd Nor, Mahasakthy Vijeyasri Satyavenathan, Nur Lina Rahmat, Alias Awang, Maizom Hassan

**Affiliations:** a Institute of Systems Biology (INBIOSIS), Universiti Kebangsaan Malaysia, 43600 UKM Bangi, Selangor, Malaysia; b Cocoa Research & Development Centre (Bagan Datuk), Malaysian Cocoa Board, P.O.Box 30, Sg. Dulang Road, 36307, Sg. Sumun, Perak, Malaysia

**Keywords:** Cocoa pod borer, *Conopomorpha cramerella*, Insect development stages, RNA-seq, Transcriptomics

## Abstract

Cocoa bean (*Theobroma cacao* L.) is part of the global cocoa and chocolate industry valued at 44 billion US dollars in 2019. Cocoa pod borer (CPB), *Conopomorpha cramerella* is a major pest of cocoa in Malaysia and Indonesia that is responsible for the decline for cocoa production. They have been detected since 1980s. Unfortunately, current control strategies are inefficient for CPB management. Although biotechnological alternatives, including RNA interference (RNAi), have been proposed in recent years to control insect pests, characterizing the genetics of the target pest is essential for successful application of these emerging technologies. We generated a comprehensive RNA-seq dataset (135,915,430 clean reads) for larva and adult stages of CPB by using the Illumina Hiseq^TM^ 4000 system to increase the understanding of CPB in relation to molecular features. The CPB transcriptome was assembled de novo and annotated. The final assembled produced 249,280 unigenes, of which 75,929 unigenes annotated against NCBI NR database and were distributed among 156 KEGG pathways. The raw data were uploaded to SRA database and the BioProject ID is PRJNA553611. The transcriptomic dataset we present are the first reports of transcriptome information in CPB that is valuable for further exploration and understanding of CPB molecular pathways.

## Specifications Table

**Table:** 

Subject	Biochemistry, genetics and molecular biology
Specific subject area	Transcriptomics
Type of data	Table Text file
How data were acquired	Illumina HiSeq™ 4000
Data format	Raw (FASTQ)
Parameters for data collection	Cocoa pod borer larvae and adults were collected from Cocoa Board Bagan Datuk Plantation, Perak, Malaysia
Description of data collection	Transcriptome of Cocoa pod borer larvae and adults
Data source location	Institution: Malaysian Cocoa Board City/Town/Region: Bagan Datuk, Perak Country: Malaysia Latitude and longitude for collected samples/data: 3.894131 N 100.8642093 E
Data accessibility	Repository name: NCBI Sequence Read Archive (SRA) Data identification number: BioProjectID: PRJNA553611 Direct URL to data: https://www.ncbi.nlm.nih.gov/bioproject/553611 Instructions for accessing these data: The raw sequence reads can be accessed from NCBI SRA with BioProjectID PRJNA553611

## Value of the Data

• The RNA-seq data obtained is from cocoa pod borer (CPB) developmental stages transcriptome. This will lead to identification of differently expressed genes between the developmental stages that will reveal putative developmental pathways and mechanisms for further exploration.
• This data will benefit molecular biology researchers of CPB in gene discovery, characterization and cloning works.
• Transcriptome data of CPB is a solid foundation for studies related to CPB development. The data is valuable for further studies on putative genes and proteins discovery that controls the development of CPB. Understanding the molecular mechanisms of CPB may lead to novel control methods of the pest.

## 1 Data Description

RNA-seq transcriptome data from CPB larvae and adult stages with three biological replicates have been obtained using the Illumina HiSeq™ 4000 sequencing platform. Raw reads from the sequencing have been uploaded to the NCBI Sequence Read Archive (SRA) database. Links and accession number to each sample fastq file is listed in Table 1. Over 135,915,430 clean reads were obtained from the Illumina sequencing. The number of reads for each sample is in Table 2. The data were also de novo assembled into full-length transcriptome (Table 3) and this can be replicated using the protocol in the methods section below.

**Table 1:** SRA accession numbers and links for raw data of CPB development transcriptome. CPB development stages: Larvae vs Adults.

Stage	Replicate	Accession number	Accession links
Larvae	Lar_L1	SRX6450311	https://www.ncbi.nlm.nih.gov/sra/SRX6450311
	Lar_L3	SRX6450312	https://www.ncbi.nlm.nih.gov/sra/SRX6450312
	Lar_L4	SRX6450313	https://www.ncbi.nlm.nih.gov/sra/SRX6450313
Adults	Adl_A2	SRX6450314	https://www.ncbi.nlm.nih.gov/sra/SRX6450314
	Adl_A3	SRX6450309	https://www.ncbi.nlm.nih.gov/sra/SRX6450309
	Adl_A4	SRX6450310	https://www.ncbi.nlm.nih.gov/sra/SRX6450310

**Table 2:** Statistics of raw and clean reads of CPB development transcriptome.

		Raw		Clean	
Stage	Replicate	Reads	Bases (G)	Reads	Bases (G)
Larvae	Lar_L1	24246778	7.3	23437855	7.0
	Lar_L3	20315775	6.1	19650804	5.9
	Lar_L4	26897022	8.1	25090045	7.5
Adults	Adl_A2	26054201	7.8	25126987	7.5
	Adl_A3	21560361	6.5	20264992	6.1
	Adl_A4	22953673	6.9	22344747	6.7

**Table 3:** Statistics of CPB development transcriptome assembly.

Attributes	Value
Number of unigenes	249,280
Percent GC	42.12 %
Average contig length	579.31 bp
Total assembled bases	144,410,072 bp

## 2 Experimental Design, Materials and Methods

### 2.1 Insect collection and rearing

Cocoa pod borer (CPB) adult and larvae were collected from cocoa pod in the Malaysian Cocoa Board Bagan Datuk Plantation, Perak, Malaysia (3.894131 N 100.8642093 E). Cocoa pods showing premature yellowing of the husk, a characteristic symptom of CPB infestation were collected in the field.

### 2.2 cDNA library construction and high-throughput sequencing

Each sample was homogenized with liquid nitrogen in a mortar and dissolved in 1 ml of TRI Reagent (Thermo Fisher Scientific) per 100 mg tissue. Total RNA was purified using RNeasy mini kit (Qiagen, Inc., Valencia, CA) following the manufacture's protocol RNA extraction with modification step. Residual genomic DNA was removed using DNA-*free*™ DNA Removal Kit (Invitrogen), according to the manufacturer's instructions. RNA quality was assessed using Nanodrop 1000 spectrophotometer (Thermo Scientific, USA). The OD_260/280_ values of each RNA sample were between 1.8 and 2.0, indicating sufficient quality. Finally, the integrity of the total RNA sample was evaluated using an Agilent 2100 Bioanalyzer (Agilent Technologies, USA), with an expected RNA integrity number (RIN) threshold of 7.0. Poly(A) RNA was isolated using the NEBNext Poly(A) mRNA Magnetic Isolation Module and libraries were prepared using the NEBNext Ultra Directional RNA Library Prep Kit for Illumina, both following the protocol of the manufacturer. Library construction and sequencing was performed by a commercial service provider Novogene Med NGS Clinical Laboratory (Tianjin, China) using Illumina HiSeq4000 at 150-bp paired-end (PE) reads (Table 1).

### 2.3 Data filtering

Weak signals and low-quality sequences were removed; read ends were also screened and trimmed for Illumina adaptor sequences by sequencing company. Approximately 142 million (142,027,810) reads were obtained, resulting in over 42.7 Gb of paired-end data. Raw reads containing ambiguous ‘N’ nucleotides (with ration of ‘N’ greater than 10%) and low quality sequences (with quality score less than 5) were removed using an in-house software (service by Novogene) in order to obtain clean read sequence (Table 2). Next, adaptor sequences were removed from the raw reads using Trimmomatic software (version 0.36) [1]. A thorough quality control on the trimmed reads was performed using FastQC software [2] written in Java to provide summary statistics for FASTQ files.

### 2.4 De novo assembly

Trinity RNA-Seq assembly software package version 2.9.1 [3] with command “Trinity –seqType fq–max_memory 80G –left [LEFT_READS_FILES] –right [RIGHT_READ_FILES] –CPU 48 –bflyCPU 12–min_contig_length 200 –full_cleanup” were used to de novo assemble the CPB transcriptome without reference genome. Transcriptome completeness was assessed by using BUSCO 4.0.6 [4] with 90.7% completeness against Insecta Odb10 and 85.7% completeness against Lepidoptera Odb10 BUSCO datasets. Trinity SuperTranscripts script were used to output the unigene sequences from the assembly (Table 3).

## Declaration of Competing Interest

The authors declare that they have no known competing financial interests or personal relationships which have, or could be perceived to have, influenced the work reported in this article.
